# Shorter Leukocyte Telomere Length in Relation to Presumed Nonalcoholic Fatty Liver Disease in Mexican-American Men in NHANES 1999–2002

**DOI:** 10.1155/2017/8435178

**Published:** 2017-06-27

**Authors:** Janet M. Wojcicki, David Rehkopf, Elissa Epel, Philip Rosenthal

**Affiliations:** ^1^Department of Pediatrics, University of California, San Francisco, CA, USA; ^2^Department of Medicine, Stanford University, Stanford, CA, USA; ^3^Department of Psychiatry, University of California, San Francisco, CA, USA

## Abstract

Leukocyte telomere length is shorter in response to chronic disease processes associated with inflammation such as diabetes mellitus and coronary artery disease. Data from the National Health and Nutrition Examination Survey (NHANES) from 1999 to 2002 was used to explore the relationship between leukocyte telomere length and presumed NAFLD, as indicated by elevated serum alanine aminotransferase (ALT) levels, obesity, or abdominal obesity. Logistic regression models were used to evaluate the relationship between telomere length and presumed markers of NAFLD adjusting for possible confounders. There was no relationship between elevated ALT levels, abdominal obesity, or obesity and telomere length in adjusted models in NHANES (OR 1.13, 95% CI 0.48–2.65; OR 1.17, 95% CI 0.52–2.62, resp.). Mexican-American men had shorter telomere length in relation to presumed NAFLD (OR 0.07, 95% CI 0.006–0.79) and using different indicators of NAFLD (OR 0.012, 95% CI 0.0006–0.24). Mexican origin with presumed NAFLD had shorter telomere length than men in other population groups. Longitudinal studies are necessary to evaluate the role of telomere length as a potential predictor to assess pathogenesis of NALFD in Mexicans.

## 1. Background

### 1.1. Nonalcoholic Fatty Liver Disease

Latinos are at high risk for specific complications from obesity including NAFLD [1] and insulin resistance compared with other population groups with the prevalence being higher in Latinos of Mexican and Central American origin than Latinos of Caribbean origin [2]. Furthermore, Latino males are at higher risk than Latino females [1].

### 1.2. Genetics and Nonalcoholic Fatty Liver Disease

Previous research has demonstrated that there is a clear genetic component (variants of the gene* PNPLA3*, I148M) to increased risk for NAFLD in Latinos of Mexican and Central American background [3]. A single nucleotide polymorphism (SNP) (rs738409) in* PNPLA*3 encodes an amino acid substitution, which results in a twofold higher liver fat accumulation among those with a GG genotype of the allele [3]. Meanwhile, however, the variant increases risk for women, not men, and Mexican and Central American origin Latinos men are at higher risk for NAFLD than women [3]. Furthermore, the addition of genetic markers to predict risk for NAFLD does not improve discriminatory ability of common clinical risk factors, suggesting the need for other biomarkers beyond genetic variants [4].

### 1.3. Telomere Length and Metabolic Disease

Cross-sectional data suggest that shorter telomere length could be a useful indicator of risk for metabolic disease. Studies in adults suggest that components of metabolic dysfunction including insulin resistance, abdominal obesity, and hypertension are associated with shorter telomere length [5]. Shorter telomere length also predicts development of type 2 diabetes mellitus [6] and progression of the metabolic syndrome [7].

In this study, we evaluate the role of leukocyte telomere length in presumed NAFLD in a population of US adults from two cycles of the National Health and Nutrition Examination Survey (NHANES) that tested leukocyte telomere length, with a focus on the relationship between telomere length and liver disease stratified by ethnic and racial background and gender.

## 2. Methods

### 2.1. Data Source and Sample

The National Health and Nutrition Examination Survey (NHANES) is a nationally representative survey of the noninstitutionalized US population. We used information from the questionnaire, laboratory, diet, and physical examination components for our analysis of the 1999-2000 through 2001-2002 surveys, as these included telomere measurements in a subset of the population aged 20 years and older that had blood collected for DNA purification. These years were the only two cycles of the NHANES that assessed telomere length. The total number of individuals was 3,567 in 1999-2000 and 4,260 in 2001-2002. All NHNAES participants 20 years old and over were asked to provide DNA samples. Of those who provided samples (10,291), 7,827 (76%) specifically allowed future DNA use and were tested for leukocyte telomere length [8]. The same laboratory, dietary, and physical exam measures were used in the two different cycles of NHANES. NHANES is approved by the National Center for Health Statistics (NCHS) Research Ethics Review Board, and written informed consent was obtained from participants. The Institutional Review Board at the University of California, San Francisco (Committee for Human Research (CHR)), exempted the present study from review.

### 2.2. Nonalcoholic Fatty Liver Disease

Our primary study outcome was the presence or absence of NAFLD. Participants were determined to have NAFLD using different indicators; if they had elevated serum alanine aminotransferase (ALT) levels (ALT ≥ 29 U/L) for men and ≥22 for women [9]. We had a secondary definition of NAFLD, which combined elevated serum ALT levels with abdominal obesity (for men > 102 centimeters and for women > 88 centimeters) [10] and a third one, which combined elevated serum ALT levels with obesity (defined using the Center for Disease Control's cut point of having a body mass index ≥ 30 kg/m^2^). Individuals who had elevated* serum ALT* levels and abdominal obesity or overall obesity were compared with participants who had neither. Those who had one indicator of presumed nonalcoholic fatty liver disease such as elevated ALT or obesity (abdominal or high body mass index (BMI)) were excluded from analyses that focused on definitions of NAFLD that included both obesity and liver enzymes so as to have a cleaner comparison group. Participants who were known to have Hepatitis B or C, HIV, and known liver disease, were currently pregnant, and had daily consumption of alcohol or known exposure to hepatotoxic medication were excluded from all analyses. Final sample size for the dataset was 7070 after excluding those with the conditions (*n* = 757).

### 2.3. Leukocyte Telomere Length Measurements

The telomere assays were conducted in the Blackburn Laboratory at the University of California, San Francisco. The Centers for Disease Control conducted a quality control review assessment before linking the leukocyte telomere length results with NHANES public files.

Briefly, aliquots of purified DNA were provided by the National Center for Health Statistics to the Blackburn Laboratory. DNA was isolated from whole blood using the Puregene (D-50K) kit protocol (Gentra Systems, Inc., Minneapolis, Minnesota) and stored at −80°C. To measure mean leukocyte telomere length, quantitative polymerase chain reaction (PCR) assay was used to determine the relative ratio of telomere repeat copy number to single-copy reference DNA number (T/S ratio) as previously described by Cawthon (2002) [11]. The single-copy gene used as a control was human beta-globin [8]. The interassay coefficient of variability for LTL was 4%.

### 2.4. Control for Potential Confounders

Potential confounders included in multivariate analysis included age in years (as continuous), age squared (based on the potential for nonlinearity between age and telomere length [12, 13]), educational attainment (less than high school, high school, or more than high school), place of birth (US born or foreign born), marital status (married or unmarried), and poverty to income ratio (the ratio of household income to poverty adjusted to family size and inflation). All variables were based on self-reported status and were found to be significant predictors for leukocyte telomere length [8]. Race/ethnicity included White, Black, Mexican-American, other Hispanic, and others or mixed race. We also adjusted for cell type composition including white blood cells (SI), lymphocytes (%), monocytes (%), neutrophils (%), eosinophils (%), basophils (%), and platelets (%), following the methodology used by Rehkopf et al. (2016) [13].

### 2.5. Statistical Analysis

Univariate and multivariate analyses were conducted using Stata 13.0 (Stata Corp, College Station, TX). We conducted stratified analyses based on sex (male versus female) and race/ethnicity (White, Black, Mexican-American, other Hispanic, and mixed race) following the designations for these years of NHANES. We fitted an interaction term to test the hypothesis that there is significant interaction between race, specifically Mexican-American background, telomere length, and presumed NAFLD as indicated by high ALT levels. As there are significantly different patterns of incidence for NAFLD based on ethnicity and gender, we conducted stratified analysis in addition to pooled analyses adjusting for the confounders described above [14, 15]. In some cases when numbers were below 100 for stratified analyses, calculations were not conducted. Sampling weights as provided for NHANES for the examination were used to take into account the complex sampling design including clustering and stratification and were incorporated into all analyses using Stata code.

A relatively small number of individuals (<15%) were missing data for the various outcomes and covariates so we decided against multiple imputation. Model 1 had less than 1% of data missing, models 2 and 3 had 13% missing, and models 4 and 5 had 14% missing.

## 3. Results

Our initial exploratory analyses assessing the possible role of race and ethnic specific differences by telomere length in predicting presumed NALFD suggested the important role of interaction. Approximately 20–30% of the participants had the outcome in question (high ALT) with slight differences based on race/ethnicity and gender (28.1% of the population with telomere length measurements had elevated ALT levels). Specifically, Mexican ethnicity interacted with telomere length indicating shorter telomere lengths for presumed NAFLD in Mexicans (OR 0.32, 95% CI 0.13–0.77) and the other/mixed race and telomere length interaction term also indicates shorter telomere length for presumed NAFLD in this population group (OR 0.07, 95% CI 0.0008–0.54) (Table 1). Adjusting for potential confounders including demographics and cell type, the interaction term for Mexican-American ethnicity became attenuated (OR 0.46, 95% CI 0.20–1.06), although it did not become attenuated for other/mixed race individuals (OR 0.03, 95% CI 0.004–0.24) (results not shown).

### 3.1. Telomere Length and Elevated ALT

In unadjusted analysis, we found that longer telomere length was associated with increased risk of elevated serum ALT levels in both sexes (OR 1.87, 95% CI 1.19–2.94) with increased risk for men in particular (OR 2.95, 95% CI 1.43–6.09) and highest risk for those who self-identified as African-American males (OR 5.52, 95% CI 1.38–22.10) and other Hispanic males (OR 17.00, 95% CI 1.67–173.24) (Table 2). Non-Hispanic White males also had increased risk based on longer telomere length (OR 3.01, 95% CI 1.32–6.91). Those that defined themselves as others or mixed/race had increased risk with shorter telomere length (OR 0.14, 95% CI 0.02–0.92) (Table 2). Mexican-Americans did not have increased risk for elevated ALT with shorter or longer telomere length (OR 0.65, 95% CI 0.31–1.35).

In adjusted analyses for demographic factors, all relationships are attenuated and lose statistical significance with the exception of mixed race/other individuals. When adjusting for cell type composition, African-American men show increased risk for elevated ALT with longer telomere length (OR 4.98, 95% CI 1.03–24.13) and mixed race/other individuals continue to have increased risk with shorter telomere length (Table 2).

### 3.2. Telomere Length and Elevated ALT and Obesity

When elevated serum ALT levels were combined with abdominal obesity or obesity, we found that shorter telomere length was associated with presumed NAFLD in Mexican-American men (OR 0.15, 95% CI 0.03–0.64 for abdominal obesity; OR 0.012, 95% CI 0.0006–0.24 for obesity) (Table 2; models 4 and 5). Mixed race/other individuals also continued to have shorter telomere length with presumed NAFLD. Similar to the prevalence of elevated ALT, the prevalence of the outcome of high ALT and high waist or obesity was similar approximately 20–30% in the population depending on race/ethnicity (20% for high ALT and high waist and 24% for high ALT and obesity in the population as a whole).

## 4. Discussion

This is the first study to assess the relationship between presumed NAFLD and leukocyte telomere length in a population-based sample of mixed race and ethnicity. Telomere length was not associated with presumed NAFLD after adjustment for sociodemographic confounders in the NHANES population as a whole. However, shorter telomere length in Mexican-origin men was significantly associated with presumed NAFLD. Importantly, there were important ethnic- and gender-specific interactions between telomere length and presumed NAFLD that were uncovered through stratified analyses.

Leukocyte telomeres are particularly sensitive to oxidative stress and inflammation [16]. Mexican-origin men are known to have increased risk for NAFLD with African-American men having reduced risk compared with whites. It is possible that shorter leukocyte telomeres may precede the inflammatory cascade that results in development of fatty liver disease and Mexican-origin men are most susceptible to the inflammatory processes resulting in excess fat deposits in hepatocytes in contrast with Caucasians [17]. Other studies have found that short telomere length contributes to the inflammatory process responsible for many divergent disease conditions [18]. Leukocyte telomere shortening in Mexican-origin men may be the initial indicator regarding development of NAFLD after elevated ALTs and obesity. Indeed, a recent study found that leukocyte telomere shortening predicted onset of NAFLD in Asian patients with type 2 diabetes mellitus [19].

Mexican-origin men may be particularly sensitive to hepatic insults and inflammation associated with obesity, while other population groups, particularly African-Americans, may be more resistant to these same physiological processes.

Indeed, studies with children have shown that insulin resistance does not have the same impact on elevated serum ALT levels in African-Americans compared with Latinos [20] and that obesity explains the association between metabolic syndrome and elevated serum ALT levels in Latinos but not in other population groups [21]. Of note, longer telomere length was associated with elevated serum ALT levels in African-American men in contrast with Mexican-Americans, although this relationship disappeared after controlling for obesity.

### 4.1. Mixed Race/Others and Shorter Telomere Length

We also found that those NHANES participants classified as others or mixed race, most commonly of Asian, Pacific Islander, or Native American background, also had shorter telomere length in relation to presumed NAFLD. There is much less information on the prevalence of NAFLD in Asian-American and Native American populations; however, it is possible that some of the Native Americans groups may have similar genetic backgrounds with Mexican-American and hence elevated risk. The “others/mixed race” category also had a small number of individuals (*n* = 197), which limits the ability to assess specific models including those including obesity and abdominal obesity. Future studies need to sample those of mixed race and Asian, Pacific Island, and Native American race in higher numbers such that strata-specific analyses are possible.

### 4.2. Further Directions

Although the NHANES dataset does test fasting laboratory values for glucose and insulin, the number of samples was small as this was done in only a subset of the larger population. Future studies should assess markers of insulin resistance including HOMA levels in addition to ALT and obesity measures as well as make use of imaging studies or histology for better diagnosis of NAFLD. Lastly, studies should further investigate the differential impact of NAFLD, inflammatory process, and progression to nonalcoholic steatohepatitis (NASH) using both leukocyte and hepatocyte telomere lengths to better understand progression of disease and differential impact based on ethnicity/race and gender.

## Figures and Tables

**Table 1:** Telomere length, race, and interaction terms on suspected nonalcoholic fatty liver disease, NHANES 1999–2002. The table included interaction terms to assess the relationship between race/ethnicity and telomere length in relation to suspected nonalcoholic fatty liver disease (defined by having elevated ALT levels).

Variable	Odds ratio (OR)	*p* value	Confidence interval
Female sex	1.03	0.85	0.75–1.40
Age in years	0.98	<0.01	0.97–0.99
Telomere length	1.25	0.50	0.63–2.48
*Race/ethnicity*			
White (non- Hispanic)	1.00		
Black (non-Hispanic)	0.50	0.29	0.13–1.87
Mexican-American	3.96	<0.01	1.73–9.04
Other Hispanic	0.54	0.50	0.08–3.89
Other/mixed race	22.02	0.01	2.04–237.90
*Interaction terms*			
MexicanXtelomere	0.32	0.01	0.13–0.77
BlackXTelomere	1.36	0.70	0.38–4.87
Other Hispanic XTelomere	2.42	0.35	0.37–15.87
Other-MixedXTelomere	0.07	0.01	0.008–0.54

**Table 2:** Logistic regression of telomere length on elevated ALT, age 20 and older, and other indicators of suspected nonalcoholic fatty liver disease, NHANES 1999–2002.

	Model 1, unadjusted	Model 2, demographic	Model 3, demographic and cell type adjusted	Model 4, demographic, cell type, and abdominal obesity	Model 5, demographic, cell type, and obesity
All races					
All sexes	**1.87 (1.19–2.94)** ^**∗∗**^ **(n = 7049)**	1.09 (0.65–1.85) (*n = *6127)	1.14 (0.67–1.94) (*n = *6115)	1.13 (0.48–2.65) (*n = *2829)	1.17 (0.52–2.62) (*n = *3373)
Male	**2.95 (1.43–6.09)** ^**∗∗**^ **(n = 3621)**	1.13 (0.49–2.61) (*n = *3195)	1.12 (0.50–2.52) (*n = *3191)	0.98 (0.29–3.24) (*n = *1607)	1.32 (0.42–4.17) (*n = *1886)
Female	1.25 (0.71–2.20) (*n = *3428)	0.91 (0.42–1.98) (*n = *2932)	0.99 (0.44–2.22) (*n = *2924)	1.087 (0.29–3.93) (*n = *1222)	0.72 (0.20–2.57) (*n = *1487)
Other Hispanic					
All sexes	**5.03 (1.05–24.13)** ^**∗**^ **(n = 312)**	4.09 (0.54–31.18) (*n = *269)	1.72 (0.15–19.44) (*n = *268)	0.74 (0.08–6.94) (*n = *128)	15.20 (0.27–860.28) (*n = *160)
Male	**17.00 (1.67–173.24)** ^**∗**^ **(n = 137)**	0.52 (0.008–34.10) (*n = *126)	0.06 (0.0005–7.80) (*n = *125)	**—**	**—**
Female	1.86 (0.27–12.75) (*n = *175)	22.51 (0.63–807.0) (*n = *143)	37.14 (0.65–2109.41) (*n = *143)	**—**	**—**
African-American					
All	2.43 (0.86–6.86) (*n = *1445)	1.86 (0.58–5.99) (*n = *1190)	1.84 (0.64–5.18) (*n = *1189)	2.09 (0.65–6.70) (*n = *479)	3.23 (0.70–14.90) (*n = *574)
Male	**5.52 (1.38–22.10)** ^**∗**^ **(n = 696)**	3.53 (0.79–15.73) (*n = *567)	**4.98 (1.03–24.13)** ^**∗**^ **(n* = *566)**	5.56 (0.16–192.8) (*n = *291)	5.84 (0.29–119.55) (*n = *315)
Female	1.34 (0.32–5.0) *n = *749	1.46 (0.27–7.75) (*n = *623)	1.28 (0.35–4.70) (*n = *623)	2.25 (0.12–42.06) (*n = *188)	2.33 (0.17–32.77) (*n = *259)
Mexican-American					
All	0.65 (0.31–1.35) (*n = *1201)	0.39 (0.14–1.09) (*n = *1007)	0.41 (0.15–1.16) (*n = *1001)	0.37 (0.09–1.52) (*n = *537)	**0.15 (0.03–0.64)** ^**∗**^ **(n* = *594)**
Male	0.46 (0.19–1.11) (*n = *537)	0.23 (0.05–1.04) (*n = *471)	0.22 (0.04–1.03) (*n = *470)	**0.07 (.006–0.79)** ^**∗**^ **(n* = *284)**	**0.012 (0.0006–0.24)** ^**∗∗**^ **(n* = *296)**
Female	1.10 (0.19–6.38) (*n* = 664)	1.03 (0.13–8.36) *n = *536	1.59 (0.22–11.54) (*n = *531)	6.00 (0.19–188.15) (*n = *253)	0.48 (0.04–5.38) (*n = *298)
Non-Hispanic White					
All	**1.93 (1.07–3.49)** ^**∗**^ **(n = 3894)**	1.136 (0.62–2.07) (*n = *3487)	1.20 (0.66–2.18) (*n = *3483)	0.98 (0.37–2.55) (*n = *1589)	0.99 (0.33–2.97) (*n = *1936)
Male	**3.01 (1.32–6.91)** ^**∗**^ **n = (2172)**	1.22 (0.50–2.96) *n = *1959	1.31 (0.54–3.16) (*n = *1958)	0.98 (0.265–3.82) (*n = *934)	1.38 (0.36–5.73) (*n = *1146)
Female	1.29 (0.56–2.97) *n* = (1722)	0.82 (0.32–2.08) *n = *1528	0.87 (0.35–2.16) (*n = *1525)	0.44 (0.11–1.91) (*n = *655)	0.37 (0.07–1.90) (*n = *790)
Others/mixed race					
All	**0.14 (0.02–0.92)** ^**∗**^ **(n = 197)**	**0.00004 (0.000000001–0.14)** ^**∗∗**^ **(n* = *174)**	**1.00e − 0.06 (2.59e − 11–0.04)** ^**∗**^ **(n* = *174)**	**1.97e − 24 (1.97e − 14–0.18)** **(n* = *96)**	**—**
Male	0.01 (0.00003–3.28) *n* = (79)	**0.00002 (1.62e − 09–0.18)** ^**∗**^ **n* = *72**	5.03*e *− 16 (8.76*e *– 45 − 2.90*e* + 13) (*n = *72)	**—**	**—**
Female	0.26 (0.04–1.64) *n* = (118)	2.85*e *− 0.08 (1.61*e* − 17–50.50) (*n = *102)	**8.46e − 08 (4.25e − 14–0.17)** ^**∗**^ **(n* = *102)**	**—**	**—**

Model 1 does not adjust for any covariates. Model 2 adjusts for the following control variables: foreign birthplace, education (less than high school, high school diploma, and more than high school), married and age (as continuous), age squared, and poverty to income ratio. Model 3 adjusts for all the variables in model 2 in addition to cell type composition, blood cells (SI), lymphocytes (%), monocytes (%), platelets (%), basophils (%), eosinophils (%), and neutrophils (%). Model 4 includes all the variables in model 3 and additionally includes abdominal obesity as an outcome (in addition to elevated ALT). Model 5 adjusts for all the variables in model 2 in addition to adding obesity as an outcome (in addition to elevated ALT). *Note*. ^*∗*^*p* < 0.05; ^*∗∗*^*p* < 0.01.
